# Optimizing Scoring and Sampling Methods for Assessing Built Neighborhood Environment Quality in Residential Areas

**DOI:** 10.3390/ijerph14030273

**Published:** 2017-03-08

**Authors:** Joel Adu-Brimpong, Nathan Coffey, Colby Ayers, David Berrigan, Leah R. Yingling, Samantha Thomas, Valerie Mitchell, Chaarushi Ahuja, Joshua Rivers, Jacob Hartz, Tiffany M. Powell-Wiley

**Affiliations:** 1 National Institutes of Health Undergraduate Scholarship Program, Office of Intramural Training and Education, Office of the Director, National Institutes of Health, Bethesda, MD 20892, USA; joel.adu-brimpong@nih.gov (J.A.-B.); samantha.thomas@nih.gov (S.T.); 2 Department of Global and Community Health, School of Public Health, George Mason University, Fairfax, VA 22030, USA; nathan.coffey4@gmail.com; 3 Donald W. Reynolds Cardiovascular Clinical Research Center at the University of Texas Southwestern Medical Center, Dallas, TX 75390, USA; colby.ayers@UTSouthwestern.edu; 4 Division of Cancer Control and Population Sciences, National Cancer Institute, Bethesda, MD 20892, USA; berrigad@mail.nih.gov; 5 Cardiovascular and Pulmonary Branch, National Heart, Lung, and Blood Institute, National Institutes of Health, Bethesda, MD 20892, USA; leah.yingling@nih.gov (L.R.Y.); valerie.mitchell@nih.gov (V.M.); chaarushi.ahuja@nih.gov (C.A.); joshua.rivers@nih.gov (J.R.); jacob.hartz@nih.gov (J.H.); 6 Division of Cardiology, Children’s National Medical Center, Washington, DC 20010, USA

**Keywords:** virtual audits, Google Street View, Active Neighborhood Checklist, built neighborhood environment, residential neighborhoods, Walk Score^®^, environment quality, Washington D.C. Cardiovascular Health and Needs Assessment

## Abstract

Optimization of existing measurement tools is necessary to explore links between aspects of the neighborhood built environment and health behaviors or outcomes. We evaluate a scoring method for virtual neighborhood audits utilizing the Active Neighborhood Checklist (the Checklist), a neighborhood audit measure, and assess street segment representativeness in low-income neighborhoods. Eighty-two home neighborhoods of Washington, D.C. Cardiovascular Health/Needs Assessment (NCT01927783) participants were audited using Google Street View imagery and the Checklist (five sections with 89 total questions). Twelve street segments per home address were assessed for (1) Land-Use Type; (2) Public Transportation Availability; (3) Street Characteristics; (4) Environment Quality and (5) Sidewalks/Walking/Biking features. Checklist items were scored 0–2 points/question. A combinations algorithm was developed to assess street segments’ representativeness. Spearman correlations were calculated between built environment quality scores and Walk Score^®^, a validated neighborhood walkability measure. Street segment quality scores ranged 10–47 (Mean = 29.4 ± 6.9) and overall neighborhood quality scores, 172–475 (Mean = 352.3 ± 63.6). Walk scores^®^ ranged 0–91 (Mean = 46.7 ± 26.3). Street segment combinations’ correlation coefficients ranged 0.75–1.0. Significant positive correlations were found between overall neighborhood quality scores, four of the five Checklist subsection scores, and Walk Scores^®^ (*r* = 0.62, *p* < 0.001). This scoring method adequately captures neighborhood features in low-income, residential areas and may aid in delineating impact of specific built environment features on health behaviors and outcomes.

## 1. Introduction

Socio-ecological models posit that the social and physical environments that we inhabit influence our health in many important ways [1,2,3,4]. For example, recent studies have linked higher neighborhood walkability [5,6], or the built environment’s ability to support walking, and Walk Score^®^ [7], a web-based neighborhood walkability measure, with increased physical activity (PA). Additionally, studies have observed associations between residing in highly walkable neighborhoods and health outcomes such as lower abdominal obesity [8], prevalence of overweight and obesity and incidence of diabetes [9]. However, the mechanism(s) linking specific aspects of the built neighborhood environment and health-related behaviors and outcomes are not well understood. With physical inactivity looming as a 21st century public health priority [10], it is imperative that we understand environmental factors that enhance and or impede PA engagement among diverse populations.

To gain insights into the neighborhood correlates of health behaviors and outcomes, recent observational studies have used omnidirectional imagery from virtual platforms such as Google Maps Street View (http://maps.google.com). These technologies, in tandem with neighborhood audit measures, have been applied to record the presence and conditions of neighborhood features such as pedestrian infrastructure and quality of environment [11]. Virtual-based neighborhood audits have been validated and observed to require less time and resources than traditional in-person, field-based audits [12,13,14,15,16,17]. However, although an acceptable means of gathering built neighborhood environment data, online-based audit tools such as Google Street View (GSV) are largely deemed first generation measures that require further optimization to maximize their utility [18]. For instance, GSV imagery alongside the Active Neighborhood Checklist (the Checklist), a validated neighborhood audit measure, has been used to audit large, urban areas and appears comparable to field-based audits [12,19]. However, little is known about optimal methods for scoring neighborhood audit measures and best street segments sampling practices when auditing specific neighborhood addresses.

Potential avenues for increasing the efficiency of neighborhood audits using virtual technology is through a more streamlined, standardized data sampling and scoring protocol [18,20,21]. McMillan et al. suggest that “*environmental audits on the complete census of streets in a neighborhood may be unnecessary, as there is likely substantial homogeneity within street types in a neighborhood, particularly residential streets.*” [21].

The goal of this study was to develop a different scoring method than previously used for the Checklist, to enable overall built environment comparisons across different neighborhoods and to be better equipped to explore both the independent and varying components of the built neighborhood environment. In addition, we aim to assess and provide researchers and other community stakeholders with information on the number of street segments needed to obtain sufficient built environment information for specific addresses in low-income, urban, residential neighborhoods. We also explore the relationship(s) between built environment quality scores and Walk Score^®^ to further evaluate the reliability and criterion validity of our scoring measure.

## 2. Materials and Methods

### 2.1. The Washington, D.C. Cardiovascular (CV) Health and Needs Assessment

The Washington, D.C. CV Health and Needs Assessment was a community-based participatory research-designed, observational study to evaluate CV health and psychosocial factors, cultural norms and neighborhood environment characteristics in a predominantly African-American population from faith-based organizations in at-risk Washington, D.C. and surrounding Maryland communities. The Washington, D.C. CV Health and Needs Assessment serves as a preliminary step in the development of a community-based behavioral change intervention to improve CV health in these communities. Further details about this study have been published previously [22,23]. This study was approved by the National Heart, Lung, and Blood Institute Institutional Review Board (ClinicalTrials.gov NCT01927783; Date of Registration: 20 August 2013). All participants provided written informed consent for the study.

### 2.2. The Active Neighborhood Checklist (the Checklist)

We paired GSV imagery with the Checklist, a validated neighborhood audit measure consisting of five sections (89 questions total). The Checklist was chosen due to its user-friendliness; it was developed to serve a broad range of audiences and designed for observation from the perspective of pedestrians [24]. Strong correlations between in-person and virtual audits using this neighborhood measure and GSV imagery have been reported in previous studies [12,19]. The Checklist is available online [25].

### 2.3. Scoring Protocol for the Checklist

In contrast to ways in which the Checklist has been utilized previously [19,26], we translated the Checklist into an Excel spreadsheet (Microsoft, Redmond, WA, USA) and scored each item on the Checklist numerically (a scale of 0–2 points per question) based on their hypothesized influence on PA engagement. In addition, 0 points were assigned to feature(s) with little to no positive effect on PA. Furthermore, 1 or 2 points conferred positive effect and presence on either one side (1 point) or both sides (2 points) of the street segment. For example, no sidewalks on a street segment received a 0 point score, 1 point for sidewalk on one side and 2 points for sidewalks on both sides of a street segment. Several items were reverse coded to keep in line with the direction of the point system (see Table S1 for audit checklist characteristics and scoring system). For each street segment, we scored the five sections of the Checklist and consolidated the scores to compute the total street segment score. An “overall” built environment quality score per address was obtained by combining the scores of the 12 street segments surrounding a participant’s address, denoting a participants’ home neighborhood or proximal immediate neighborhood (see Table 1 for maximum possible audit scores).

### 2.4. Virtual Audit Training Procedure

Three research assistants at the National Institutes of Health engaged in a 1-hour training session introducing the GSV tool and the Checklist. The purpose of the brief training session was to assess immediate impressions and experiences with the tools and to identify discrepancies between trainees’ interpretations. Trainees conducted initial audits, blinded to each other’s audit results, followed by group discussion on problems incurred during audits. After training, a graduate student intern, one of the three assistants, conducted the audits used in this study. Six participants’ addresses were randomly selected for a second-look audit by another research assistant, blinded to the results of the first audits. Inter-rater reliability was assessed for the current study.

### 2.5. Conducting Virtual Neighborhood Audits

**Figure 1:**
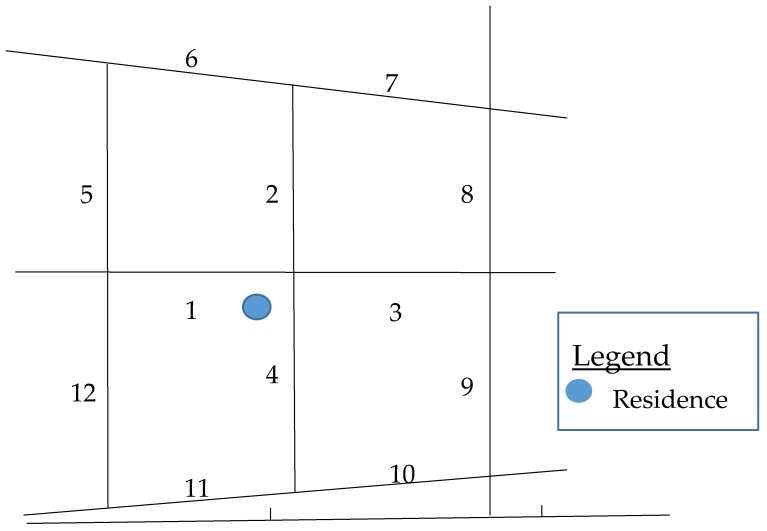
This is an example of a neighborhood segment map that would be created for each participant before virtual audits. The lines denote different streets, forming street intersections where lines intersect. The blue dot denotes a participant’s residence. The neighborhood around a specific address, or the proximal immediate neighborhood, is defined as the 12 closest street segments to a specific address. Street segment is consistent with established definitions of where one street intersects another.

Eighty-two home addresses of study participants living in Washington, DC and surrounding Maryland areas were obtained as part of the Washington, D.C. CV Health and Needs Assessment (see Figure S1 for neighborhood audit regions map). GSV imagery and the Checklist were used for virtual audits. Up to 16 street segments, approximately 4 blocks in length, immediately adjacent to participants’ home addresses, were assessed for (1) Land-Use Type; (2) Public Transportation Availability; (3) Street Characteristics; (4) Environment Quality and (5) Sidewalks for walking, biking and related features (the five sections of the Checklist). A neighborhood streets segment map was created for each address (illustrated in Figure 1). Furthermore, 972 total street segments were audited; and 948 were included in this study as only the 12 closest street segments for each address were available for all study participants.

### 2.6. Walk Score^®^

Walk Score^®^ is a validated, publicly available, web-based neighborhood walkability tool originally designed for real estate purposes to assess the number of nearby amenities or walkable destinations [28,29,30,31]. Using a geographically-based algorithm and data provided by the Google AJAX Search application interface, Walk Score^®^ assigns neighborhood walkability score to an area based on the availability of and proximity to each of 13 different amenity categories: grocery stores, coffee shops, restaurants, bars, movie theatres, schools, parks, libraries, book stores, fitness centers, drug stores, hardware stores, and clothing/music stores [32]. The Walk Score^®^ point system ranges from 0 to 100 with varied weights for amenity categories. The points are summed and normalized [28,29,32]. Several studies examining the influence(s) of neighborhood walkability on PA behaviors have been published using this tool [7,33]. As a further evaluative measure of our scoring protocol, we obtained Walk Scores^®^ of our participants’ home addresses from [27]. We examined correlation(s) between study participants’ virtual audit scores and Walk Scores^®^ using Spearman calculations as an additional measure to further evaluate the reliability of our scoring method.

### 2.7. Statistical Analysis

Using a code generated in Statistical Analysis Software (SAS version 9.2, SAS Institute, Inc. Cary, NC, USA), we tested all potential combinations of street segments to determine how participants’ street segment scores compare with their overall built neighborhood environment quality score (see Figure S2 for example of coding). Because the goal of this step of the study was to assess the minimum number of street segments needed to gain adequate information about participants’ built neighborhood environment, we ran a statistical code that generated street segment mean scores from the 12 street segment scores per address. For example, the combinations coding for randomly selecting four street segment scores out of 12 street segment scores, or _12_C_4_, simulated all possible ways in which 4 street segment scores could be randomly chosen from the 12 street segment scores per address (*n* = 495), without any repeated segment combinations (i.e., each combination is unique). The code would then calculate a mean for the _12_C_4_ combinations. Again, the means were generated for all possible street segment combinations starting from one street segment up to 12 street segments combinations (i.e., _12_C_1_, _12_C_2_…_12_C_12_), per address, in order to evaluate the cumulative effects of each additional street segment score added. Participant combination means were then averaged together across all participants to obtain overall combination means (i.e., overall means for _12_C_1_, _12_C_2_…_12_C_12_). Spearman’s rank-based correlation coefficients were generated to determine the relationship between the segment combination means (i.e., _12_C_1_, _12_C_2_…_12_C_12_) and each participant’s overall mean. Analyses were performed for the overall population and separate analyses were conducted for participants living in Washington D.C. and in Maryland. There was little difference between findings for the overall population and findings for those living in Washington, D.C. or Maryland separately; therefore, only findings for the overall population are shown here.

### 2.8. Inter-Rater Reliability Coefficient

Inter-rater reliability was assessed by calculating a prevalence-adjusted bias-adjusted kappa (PABAK) coefficient. PABAK has been used in prior built environment studies to assess inter-rater reliability [12,19,34] and takes into account systematic differences between data sources and low variability in distribution of audit items [12,35]. The PABAK coefficient for our study was 0.88, denoting a high inter-rater reliability agreement using Landis and Koch-based adjectival rating interpretations [36].

## 3. Results

Street segment quality scores ranged 10–47 (Mean = 29.36 ± 6.87). Overall built environment quality scores, or neighborhood quality scores, for the five sections of the Checklist ranged: (1) Land-Use Type (26–83 points, Mean = 50.96 ± 14.51); (2) Public Transportation Availability (0–15 points, Mean = 2.73 ± 3.52); (3) Street Characteristics (23–62 points, Mean = 41.05 ± 10.07); (4) Environment Quality (39–97 points, Mean = 65.98 ± 11.93); and (5) Sidewalks/Walking/Biking features (53–258 points, Mean = 191.60 ± 44.41). Overall neighborhood quality scores ranged from 172 to 475 (Mean = 352.32 ± 63.55) (see Table 1 for maximum possible scores).

**Figure 2:**
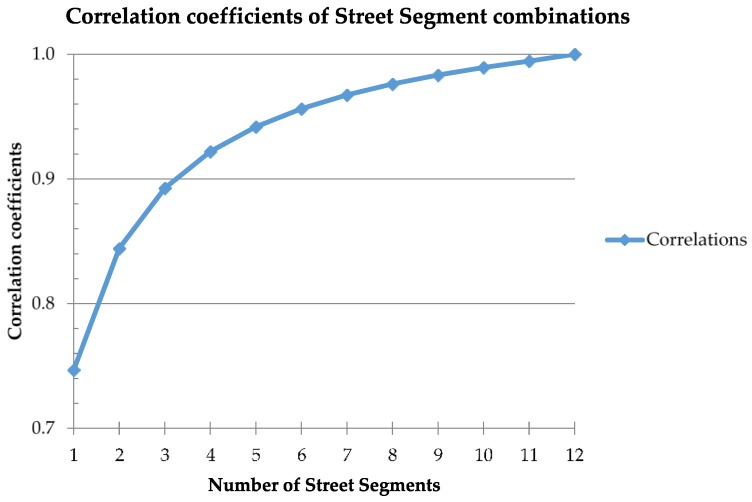
Correlation coefficients obtained from street segment combinations coding. Combination coefficients were generated by code developed in Statistical Analysis Software which runs a series of combinations of scores from one street segment up to twelve segments (i.e., _12_C_1_, _12_C_2_…_12_C_12_) per participant, simulating all possible ways in which a certain number of street segment scores can be randomly chosen from the overall 12 street segment scores, without any repeated segment combinations. The code calculated means for the selected combinations then averaged the means across all participants to obtain overall combination means (i.e., overall means for _12_C_1_, _12_C_2_…_12_C_12_). Spearman calculations used to determine associations between overall segment combination means (i.e., _12_C_1_, _12_C_2_…_12_C_12_) and participants’ segment means.

The correlation coefficients obtained from the random selection of street segment combinations ranged from 0.75 to 1.00 (see Figure 2), with *r* = 0.89 for any three randomly-selected street segments scores per address. Participants’ neighborhood Walk Score^®^ ranged from 0 to 91 (Mean = 46.65 ± 26.29). Significant positive correlations were found between overall built environment quality scores and Walk Scores^®^ (*r* = 0.62, *p* < 0.001). Four of the five main categories of the built neighborhood environment features, (i.e., Land-Use Type (*r* = 0.70, *p* < 0.0001), Public Transportation Availability (*r* = 0.33, *p* = 0.003), Street Characteristics (*r* = 0.55, *p* < 0.0001) and Sidewalk/Walking/Biking Features (*r* = 0.57, *p* < 0.0001)), showed significant correlations with Walk Scores^®^. No significant correlation was observed between Environment Quality (*r* = −0.20, *p* = 0.07) and Walk Scores^®^.

## 4. Discussion

The focus of the present study was to develop a scoring method for the Checklist and to determine the minimum number of street segments needed to obtain sufficient built environment information for specific neighborhood addresses in low-income, urban, residential areas utilizing virtual audits. Our results suggest that the proposed scoring method for the Checklist is a reliable means of obtaining neighborhood built environment quality information for specific neighborhood addresses. Additionally, we observed that virtual audits of three street segments in the immediate vicinity of homes in low-income, urban, residential areas provide sufficient data on the built neighborhood environment quality in the area. However, we advise researchers and community stakeholders to use this information as guide posts when conducting neighborhood-based studies or taking community actions. Lastly, we demonstrate the reliability of virtual audits utilizing the proposed scoring method with Walk Score^®^, a validated neighborhood walkability tool. This study responds to requests for the development of simple, yet effective scoring and sampling methods for assessing built neighborhood environment quality in residential areas and adds to the emergent literature of virtual-based neighborhood audits and Walk Score^®^.

The validity and reliability of the Checklist has been well established [12,19,24,26]. Consistent with methods employed in previous studies utilizing this neighborhood audit measure, we recorded the presence and/or absence of neighborhood micro-environmental features on both sides of residential street segments [12,19,24,26]. However, the aforementioned studies, in their analyses, dichotomized all items on the Checklist as present (on one or both sides of the street segment) or absent. For example, sidewalks were analyzed dichotomously as present (on one or both sides of street segment) or absent. For the current study, we quantified the presence (1 point on one side and 2 points on both sides of street segment) or absence (0 points) of sidewalks (see Table S1). A recent paper by Frackelton et al. [37] speaks to the need and importance of detailed, high-quality data regarding pedestrian infrastructure in the planning and creation of accessible pedestrian facilities. Such information as captured by the proposed scoring method could aid in providing granular data (e.g., understanding how the availability of sidewalks on one side or both sides of the street is utilized and in what neighborhoods one sidewalk may prove more cost-effective than others, etc.) to inform effective resource allocation for infrastructure investment in different neighborhoods. By quantifying all of the Checklist items, we aim to be better equipped to explore both the independent and varying components (i.e., Land-Use Type, Public Transportation Availability, Street Characteristics, Environment Quality, Sidewalks/related features) of the built neighborhood environment and the potentially synergistic relationship between overall built neighborhood environment quality and observed health behaviors and outcomes.

This scoring technique may aid in delineating the influences of singular and aggregate neighborhood built environment factors on observed and measured health-related behaviors across multiple layers of analyses. In a 2013 methods study, Millstein et al. [20] used similar scoring processes (0/1 points) for dichotomously coded items (no/yes) and 0–2 points for frequency items on the Micro-scale Audit of Pedestrian Streetscapes (MAPS) audit measure. A 2014 follow up study by Cain et al. [38] utilized the MAPS scoring method to peruse the effects of the built neighborhood environment on PA engagement. The authors observed that the strongest associations between the neighborhood micro-environment and PA were with both independent (assessed as numerical subsection scores) and overall built neighborhood environment scores (comprised of the summation of subsection scores). The ability of the MAPS measure to quantify neighborhood environment features greatly aided in the capacity to draw these conclusions. The authors conclude that the independent and synergistic associations observed between the built neighborhood environment and PA suggests that PA behavior is likely impacted by the cumulative nature of various environmental attributes that band to create supportive environments for health behaviors [38]. Our proposed scoring method aids in both the disaggregation and aggregation of neighborhood features in a quantified manner that makes for similar analyses for studies employing the Checklist. As we continue to learn more about the influences of certain features in neighborhoods and their potential impact on health-related behaviors, as well as the minimum number of features that may need to be present on one side or both sides of street segments to have an intended behavioral effect, treating the presence of certain features as dichotomous variables may be problematic as it may mask the subtle differences in neighborhoods that may give rise to observed health behaviors. These subtle differences may be crucial in contributing to the overall appeal of an environment for PA engagement.

Additionally, we observed that three street segments, around specific residential addresses in low-income, urban areas, provide adequate information on the built neighborhood environment quality in the immediate vicinity, or “proximal immediate neighborhood” [21,39]. This finding corroborates previous studies that have investigated the representativeness of street segments in residential neighborhoods [21,39]. In a 2010 study, McMillan et al. [21] examined 11 low-income housing development areas in Houston, TX and 50 to 301 street segments per neighborhood, finding that 25% of street segment samples were comparable to 50%, 75% and 100% of the street segments within a 400 m buffer of these low-income housing developmental areas. We observed a similar trend in our findings from built neighborhood environment quality audits in low-income neighborhoods in Washington, DC and surrounding Maryland areas. Of the 12 closest street segments clustered around a study participant’s residence, we found that randomly selecting any three, or 25%, of the street segments captured nearly 90% of the built neighborhood environment information in the vicinity. The trend increases with each additional street segment such that at six, or 50%, of randomly selected street segments neighboring an individual’s home address, we captured 96% of the built micro-environment features of the neighborhood. Although intuitive that large similarities may exist between residential street segments in specific geographic regions, likely due to uniformity of the urban structure in the U.S., we demonstrate this relationship empirically via virtual audits.

Two prior studies sought to answer the extent in which samples of street segments provide efficient and representative overview of the overall built neighborhood environment thus far [21,39]. However, both studies employed in-person, field-based observational audit techniques. The present study is timely and important in that it expands previous findings to virtual audits as the field moves towards virtual-based neighborhood environment audits. Moreover, McMillan’s study was carried out in housing developments, meaning that the area was likely being developed in a similar manner, thereby limiting its generalizability. Cerin et al.’s [39] study was not in the U.S. Additionally, these two studies reported percentage-based findings that may constitute different applications for study areas with a larger or smaller number of street segments. For example, studies sampling at 25% or 50% of 50 street segments versus 150 street segments would yield different numbers of street segments for analyses. Our study reports the minimum numerical value, rather than percentage, of street segments in low-income, urban centers that must be audited to obtain sufficient generalizable neighborhood built environment information. Furthermore, McMillan’s analyses were based on five key variables: sidewalk presence, ratings of attractiveness, safety for walking, connectivity and number of traffic lanes. Although important, these factors do not constitute the totality of factors in neighborhoods with potential health implications. Our findings are based on a more encompassing audit measure. However, we caution against simply using three street segments around a residence to draw substantial conclusions about neighborhoods. Instead, we present data on the number of street segments clustering an address in low-income, urban communities and the degree of information they capture. We leave it to the discretion of researchers and community stakeholders to use and choose error tolerance levels in their planning, research, analyses and actions.

Lastly, our study is the first comparison of GSV micro-environmental audits and Walk Score^®^. Our findings indicate the ability of our proposed novel scoring method to capture built neighborhood environment quality information corroborated by a validated neighborhood walkability public health tool. Specifically, we found that Land-Use Type, Public Transportation Availability, Street Characteristics and Sidewalks/related features are quality measures significantly associated with Walk Score^®^. These findings add to previous reports of virtual audits [16,19,24,40,41] and Walk Scores^®^ [29,30,31] as validated and reliable measures of the neighborhood environment. More importantly, these findings demonstrate the ability of our novel scoring method to document and capture important built neighborhood environment quality information. In a prior study, Carr et al. [28] assessed correlation(s) between the built neighborhood macro-environment (i.e., street connectivity, residential density, public transit availability) and Walk Score^®^. Similarly, we found observed significant associations between Walk Score^®^ and public transit availability. Lastly, we observed that, although built neighborhood environment quality and neighborhood walkability are well correlated, virtual audits utilizing GSV imagery appear to provide additional insights beyond neighborhood walkability information provided by the Walk Score^®^ platform. Agreeably, one of the main critiques of Walk Score^®^ has been its inability to dissociate the quality of amenities in neighborhoods from their presence [28]. Walk Score^®^ provides a rough estimate of the walkability of an area based on the number of amenities present but does not provide specificities such as quality (e.g., healthy vs. less healthy eateries) [28]. Lending credibility to this critique, we found no significant correlation between environment quality subsection scores and Walk Score^®^. Virtual-based audits have the capacity to display and capture the density and quality of amenities in an environment, allowing researchers to scrutinize different aspects of the built environment to better elucidate environmental determinants of health-related behaviors and outcomes.

### 4.1. Strengths

Our study is unique in utilizing virtual audits to assess “how many segments an auditor must walk” [21] to obtain sufficient built environment information in residential settings. The strengths of the current study include: (1) the use of virtual tools to assess the built neighborhood environment quality around specific residential addresses; (2) the development of a simple scoring method for virtual audits utilizing the Checklist and (3) the provision of empirical data to support different street segment sampling practices for specific home address audits. Understanding individual and aggregate components, especially of specific neighborhood environments, is essential in informing a more tailored and adaptive intervention approach for residents of particular neighborhoods. Brownson and colleagues [18], as well as Millstein et al. [20], highlight the importance of conducting address specific neighborhood audits to better understand the features in the neighborhood most salient to the observed health-related behaviors of individuals, an approach that may provide more effective, tailored, place-based health behavior intervention [42].

### 4.2. Limitations

Our study is not without its limitations. The current study is limited to low-income, urban, residential areas in Washington, DC and surrounding Maryland areas. Table 1 depicts extremely low street segments, overall neighborhood quality and Checklist subsection scores for the residential neighborhoods audited compared with the maximum scores possible. Although this finding makes sense in context of the predominantly low-income, low-resource DC and Maryland neighborhoods inhabited by our study participants, it is possible that this could have affected our analyses. Thus, our findings may not be generalizable to rural, or affluent neighborhoods, as well as countries outside of the U.S. Additionally, GSV imagery coverage has mostly been available in urban areas in Canada, United States, Europe, Australia and parts of Asia [17]. Efforts, however, are underway to expand and improve such open-source mapping technologies to other areas of the world. Virtual audits, using such tools, then have the potential to aid researchers globally in mapping built environment onto health. Furthermore, we used Walk Score^®^, a virtual walkability measure, as a criterion measure to compare the proposed scoring method. Although in-field audits are preferable, comparing our virtual-based scoring measure with in-field audits would not necessarily aid in validating the reliability of the proposed scoring protocol itself. Instead, the focus would be on comparing virtual audit methodology utilizing the Checklist and in-person audits, which has already been demonstrated [19,26]. Walk Score^®^ is appropriate for this study as it is a valid, neighborhood environment quality measure that reports microscale data numerically, a feature essential to gauging the effectiveness of the proposed numerical scoring system. Several studies examining the influence(s) of neighborhood walkability on PA behaviors have already been published using this tool [7,33]. However, we recognize that Walk Score^®^ only captures certain aspects of the neighborhood built environment (i.e., access to local shops and services), while the Checklist summary score is much more comprehensive (e.g., covering other aspects such as pedestrian infrastructure). This is likely why we observed significant findings between parts of the Checklist scores (i.e., land-use type and sidewalk and related features) and Walk Scores^®^ and not features such as quality of environment, an aspect of the environment not captured by Walk Score^®^. This, in itself, is informative that we captured important aspects of the built environment and that utilizing virtual audits garners more information than relying solely on Walk Score^®^. Lastly, we recognize that we did not assign weights to items or categories of the Checklist in this study, which likely impacts study outcomes. Because the Checklist categories contain different quantities of built environment features/items, the scoring system is inherently influenced by categories of larger numbers of total items (e.g., street characteristics vs. public transportation). As the field of virtual audits is a nascent field, we currently do not have enough data on the impact of specific built environment features to assign weights. The Checklist includes the most important features/items of the built environment that can be measured whether in-person or via virtual audits.

## 5. Conclusions

This study provides a novel scoring protocol for studies utilizing GSV imagery and the Checklist that may help deconstruct the benefits associated with the presence or absence of certain neighborhood built environment features without condensing these features into dichotomous variables. Although developed with the Checklist, our scoring method may be applicable to other neighborhood audit measures as these measures are increasingly converging to measure similar neighborhood features for more standardized and comparative analyses across studies. It is also the first study, to our knowledge, to assess how many street segments are needed to gain sufficient insights into low income, urban, residential neighborhoods using virtual audits. Our findings aid in answering requests for more standardized sampling and scoring methods for virtual-based neighborhood audits and have implications for expediting the neighborhood built environment audit process around specific addresses. Ultimately, evaluating neighborhood built environment quality may aid in characterizing the relationship between micro-environment features with health outcomes in a specific community and subsequently designing effective, tailored, place-based interventions for improved health.

## Figures and Tables

**Table 1:** Results of Google Street View-based audits utilizing the Active Neighborhood Checklist and new scoring method.

Audit Features	Maximum Score	Range of Observed Scores	Mean (SD) Scores
^‡^ Per Street Segment	87	10–47	29.36 (6.87)
* Audit Total (overall neighborhood)	1044	172–475	352.32 (63.55)
A. Land-Use Type	372	26–83	50.96 (14.51)
B. Public Transit	48	0–15	2.73 (3.52)
C. Street Characteristic	144	23–62	41.05 (10.07)
D. Quality of Environment	144	39–97	65.98 (11.93)
E. Sidewalk Features	336	53–258	191.60 (44.41)
Walk Score^®^	100	0–91	46.65 (26.29)

^‡^ Per Street Segment denotes the score for a single street segment (i.e., one street segment out of the 12 street segments per address); * The Audit Total (overall neighborhood) score is the sum of scores from the 12 street segments audited per address. A, B, C, D and E are sub-scores of the Audit Total score. Maximum score indicates the maximum possible points per category. Range of observed scores indicates scores observed from participants’ neighborhood virtual audits. Street segment, Audit Total and sub-scores, A, B, C, D, E, were obtained from virtual audits using Google Maps Street View imagery, the Active Neighborhood Checklist and the new scoring paradigm of assigning 0, 1 or 2 for the presence or absence of built environment features. Walk Scores^®^ were obtained online [27].

## Supplementary Materials

The following are available online at www.mdpi.com/1660-4601/14/3/273/s1, Figure S1: Map of Washington D.C and Prince George’s County/Maryland neighborhood audit regions, Figure S2: Short code developed in SAS for twelve choose 5, or _12_C_5_, street segment combinations and Table S1: Audit checklist characteristics, sections and scoring procedure. Additionally, we acknowledge the importance of making research data publically available. The data analyzed during the current study is available from the corresponding author upon reasonable request.
