# Supplementary material for: Optimizing Scoring and Sampling Methods for Assessing Built Neighborhood Environment Quality in Residential Areas
Source: Int J Environ Res Public Health. 2017 Mar 8;14(3):273. doi: 10.3390/ijerph14030273 (PMC5369109; doi:10.3390/ijerph14030273)
Supplement: Supplementary file 1 [file ijerph-14-00273-s001.pdf]

**Table S1.** The audit checklist characteristics, sections and scoring procedure.

| Audit Checklist Characteristics | Possible Score | | |
|---|---|---|---|
| | 0 | 1 | 2 |
| **A. Land-Use Type** | | | |
| *1. Are residential and non-residential land uses present? **Select one*** | | | |
| All residential | Yes | | |
| Both Residential and non-residential | | Yes | |
| All non-residential | | Yes | |
| *2. What is the predominant land use? **Check one or two that apply*** | | | |
| Residential buildings/yards | | Yes | |
| Commercial, institutional, office or industrial building(s) | | Yes | |
| School/school yards (elementary, middle, high school) | | Yes | |
| Parking lots or garages | Yes | | |
| Park with exercise/sport facilities or playground equipment | | Yes | |
| Abandoned building or vacant lot | Yes | | |
| Undeveloped land | Yes | | |
| Designated green space (including park with no exercise/play facilities) | | Yes | |
| *3. What types of residential uses are present? **Select all that apply*** | | | |
| None | Yes | | |
| Abandoned homes | Yes | | |
| Single family homes | | Yes | |
| Multi-unit homes (2-4 units) | | Yes | |
| Apartments or condominiums (1-4 stories) | | Yes | |
| Apartments or condominiums (>4 stories) | | Yes | |
| Apartment over retail | | Yes | |
| *4. What functioning parking facilities are present? **Select all that apply*** | | | |
| None (no parking allowed on street most or all of the time) | | Yes | |
| Personal driveway and/or parking garage | Yes | | |
| On-street, including angled parking | | Yes | |
| Small lot or garage (<30 spaces) | Yes | | |
| Medium to large lot | Yes | | |
| Multi-Level Parking Garage Structure | Yes | | |

| | | | |
|---|---|---|---|
| *5. What public recreational facilities and equipment are present (including in the schoolyard if publically accessible)? Any features visible should be included.* ***<u>Select all that apply</u>.*** | | | |
| None | Yes | | |
| Park/Open field | | Yes | |
| Off-road walking/biking trail | | Yes | |
| Sports/playing field | | Yes | |
| Basketball/tennis/volleyball court | | Yes | |
| Playground | | Yes | |
| Outdoor pool | | Yes | |
| *6. (OPTIONAL) What types of non-residential uses are present?* ***<u>Select all that apply</u>.*** | | | |
| None | Yes | | |
| Abandoned building or vacant lot | Yes | | |
| Small grocery, convenience store (including in gas station), or pharmacy | | Yes | |
| Supermarket | | Yes | |
| Food establishment (restaurant, bakery, café, coffee shop, bar) | | Yes | |
| Entertainment (e.g., movie theatre, arcade) | | Yes | |
| Library or post office | | Yes | |
| Bank | | Yes | |
| Laundry/dry cleaner | | Yes | |
| Indoor fitness facility | | Yes | |
| School (elementary, middle, high school) | | Yes | |
| College, technical school, or university | | Yes | |
| High-rise building (>5 stories) | | Yes | |
| Big box store (e.g., Walmart, Office Depot, Best Buy) | | Yes | |
| Mall | | Yes | |
| Strip mall | | Yes | |
| Large office building, warehouse, factory, or industrial building | | Yes | |
| Church | Yes | | |
| **Total** | **0** | **31** | |

| **B. Public Transportation Availability** | | | |
|---|---|---|---|
| 1. Any transit stop (bus, train, or other)? | No | Yes, one side | Yes, both sides |
| **1a.** Bench or covered shelter at transit stop? | No | Yes, one side | Yes, both sides |
| **Total** | **0** | **2** | **4** |

| C. Street Characteristics | | | |
|---|---|---|---|
| **1.** Enter posted speed limit: | **N/A or >30** | **10-30 MPH** | |
| **2.** Enter special speed zone: | **N/A or >30** | **10-30 MPH** | |
| **3.** Enter total # of lanes on street: | **>4 lanes** | **1-4 lanes** | |
| **4.** Marked lanes? | **No** | **Yes** | |
| **5.** Median or pedestrian island? | **No** | **Yes** | |
| **6.** Turn lane? | **No** | **Yes** | |
| **7.** Stop sign or light crossing this segment? | **No** | **Yes** | |
| **7a.** Any stoplight(s) without a walk signal? | **Yes** | **No** | |
| **8.** Crosswalk for crossing this segment? | **No** | **Yes** | |
| **9.** Traffic calming device (roundabout, speed bump, brick road, other)? | **No** | **Yes** | |
| **10.** Cul-de-sac (dead-end street)? | **No** | **Yes** | |
| **10a.** Sidewalk cut-through in cul-de-sac? | **No** | **Yes** | |
| **Total** | **0** | **12** | |

| D. Environment Quality | | | |
|---|---|---|---|
| **1.** Any commercial buildings adjacent to the sidewalk? | **No** | **Yes** | |
| **2.** Any pedestrian amenities? | | | |
| **2a.** Bench (excluding at transit stop)? | **No** | **Yes** | |
| **2b.** Drinking fountain? | **No** | **Yes** | |
| **2c.** Pedestrian-scale lighting? | **No** | **Yes** | |
| **3.** Public art (e.g., statues, sculptures)? | **No** | **Yes** | |
| **4.** Graffiti or broken/boarded windows? | **Yes** | **No** | |
| **5.** Litter or broken glass? | **A lot** | **Some** | **None or a little** |
| **6.** Tree shade on the walking area? | **None or a little** | **Some** | **A lot** |
| **7.** Steepest slope along walking area? | **Steep** | **Moderate** | **Flat/gentle** |
| **Total** | **0** | **9** | **12** |

| E. Sidewalks/Biking/Walking features | | | |
|---|---|---|---|
| *SIDEWALKS* | | | |
| 1. Sidewalk present? If "No" mark "N/A" for questions 2-8 and answer 9a and 9b. | No | Yes, one side | Yes, both sides |
| 2. Any grassy or other buffer between curb and sidewalk along most of the segment? | No | Yes, one side | Yes, both sides |
| 2a. Tree(s) in buffer? | No | Yes, one | Yes, both sides |

| | | | |
|---|---|---|---|
| | | side | |
| 3. Sidewalk continuous? | No | Yes, one side | Yes, both sides |
| 4. Width >3 ft. for most of the sidewalk? | No | Yes, one side | Yes, both sides |
| 5. Width <3 ft. for any part of the sidewalk? | Yes, both sides | Yes, one side | No |
| 6. Any missing curb cuts or ramps at intersections or driveways? | Yes, both sides | Yes, one side | No |
| 7. Any major bumps, cracks, holes, or weeds in the sidewalk? | Yes, both sides | Yes, one side | No |
| 8. Any permanent obstructions (trees, signs, tables) blocking the 3-ft walk area? | Yes, both sides | Yes, one side | No |
| ***9. *Answer ONLY:*** *If a sidewalk is not present on any part of the segment, do you have another safe place to walk, including:* | | | |
| *9a. Street or shoulder (if safe)?* | *No/NA* | *Yes, one side* | *Yes, both sides* |
| *9b. Unpaved pathway?* | *No/NA* | *Yes, one side* | *Yes, both sides* |
| *SHOULDERS (OPTIONAL)* | | | |
| 10. Designated bike route sign or marking or "Share the Road" sign? | No | Yes, one side | Yes, both sides |
| 11. On-street, paved, and marked shoulder? If "No" mark "N/A" for questions 12-14 and answers 15a and 15b. | No | Yes, one side | Yes, both sides |
| 12. Width of marked shoulder ≥ 4ft? | No/NA | Yes, one side | Yes, both sides |
| 13. Shoulder continuous? | No/NA | Yes, one side | Yes, both sides |
| 14. Any permanent obstructions in the shoulder (including drainage grates, parked cars)? | Yes, both sides | Yes, one side | No |
| **15. **Answer ONLY:** If a paved, marked shoulder is not present, do you have another safe place to bicycle, including:* | | | |
| *15a. Street?* | *No/NA* | *Yes, one side* | *Yes, both sides* |
| *15b. Wide outside lane (~15ft)?* | *No/NA* | *Yes, one side* | *Yes, both sides* |
| **Total** | **0** | **14** | **28** |

*87 points is the maximum possible score per segment. Total possible score per neighborhood is 1044 points (87pts x 12 segments). 87 points is the maximum score because two items, E9 and E15, need not be answered if sidewalks or shoulders are present on any part of the segment. Additionally, some of the checklist items (e.g., items 1 and 2) require that you select only one or two of the item choices.

**Figure S1.** Map of Washington D.C and Prince George's County/Maryland neighborhood audit regions.

## Neighborhood Audit Regions

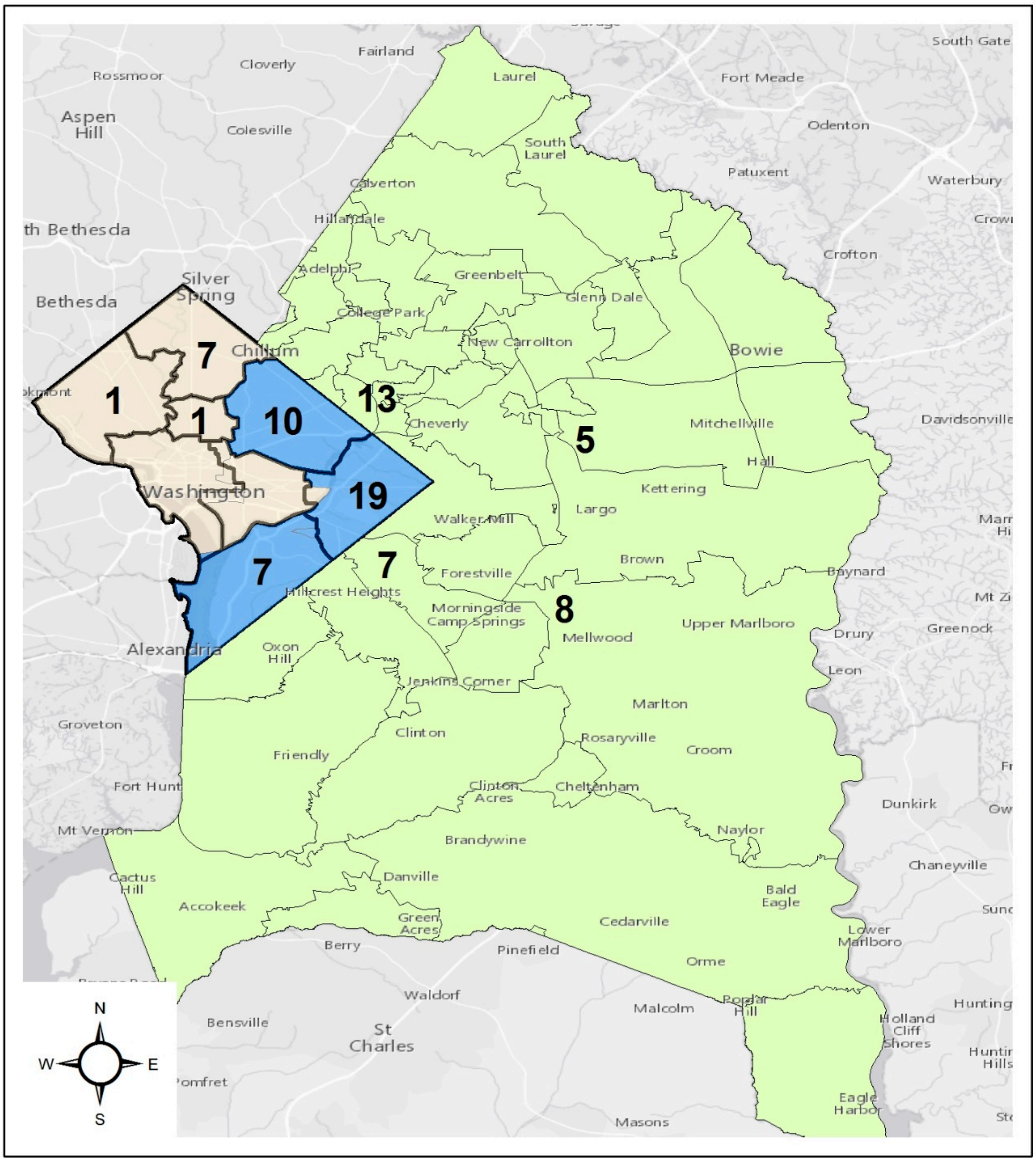


**Notes:**

- Blue depicts Washington D.C., wards 5, 7 and 8
- Green depicts surrounding Prince George's County/Maryland (MD) areas, where majority of audits in MD occurred
- The numbers represent number of different households audited in the general area
- There are 82 participants total; 4 participants' addresses fell outside of the highlighted areas

**Figure S2.** Short code developed in SAS for twelve choose 5, or $_{12}C_{5}$, street segment combinations.

```
“…
data complete;
set complete;
if combo1='Segment1' then score1=segment1;
if combo1='Segment2' then score1=segment2;
if combo1='Segment3' then score1=segment3;
if combo1='Segment4' then score1=segment4;
if combo1='Segment5' then score1=segment5;
if combo1='Segment6' then score1=segment6;
if combo1='Segment7' then score1=segment7;
if combo1='Segment8' then score1=segment8;
if combo1='Segment9' then score1=segment9;
if combo1='Segment10' then score1=segment10;
if combo1='Segment11' then score1=segment11;
if combo1='Segment12' then score1=segment12;

if combo2='Segment1' then score2=segment1;
if combo2='Segment2' then score2=segment2;
if combo2='Segment3' then score2=segment3;
if combo2='Segment4' then score2=segment4;
if combo2='Segment5' then score2=segment5;
if combo2='Segment6' then score2=segment6;
if combo2='Segment7' then score2=segment7;
if combo2='Segment8' then score2=segment8;
```

```
if combo2='Segment9' then score2=segment9;
if combo2='Segment10' then score2=segment10;
if combo2='Segment11' then score2=segment11;
if combo2='Segment12' then score2=segment12;

if combo3='Segment1' then score3=segment1;
if combo3='Segment2' then score3=segment2;
if combo3='Segment3' then score3=segment3;
if combo3='Segment4' then score3=segment4;
if combo3='Segment5' then score3=segment5;
if combo3='Segment6' then score3=segment6;
if combo3='Segment7' then score3=segment7;
if combo3='Segment8' then score3=segment8;
if combo3='Segment9' then score3=segment9;
if combo3='Segment10' then score3=segment10;
if combo3='Segment11' then score3=segment11;
if combo3='Segment12' then score3=segment12;

if combo4='Segment1' then score4=segment1;
if combo4='Segment2' then score4=segment2;
if combo4='Segment3' then score4=segment3;
if combo4='Segment4' then score4=segment4;
if combo4='Segment5' then score4=segment5;
if combo4='Segment6' then score4=segment6;
if combo4='Segment7' then score4=segment7;
```

```
if combo4='Segment8' then score4=segment8;
if combo4='Segment9' then score4=segment9;
if combo4='Segment10' then score4=segment10;
if combo4='Segment11' then score4=segment11;
if combo4='Segment12' then score4=segment12;

if combo5='Segment1' then score5=segment1;
if combo5='Segment2' then score5=segment2;
if combo5='Segment3' then score5=segment3;
if combo5='Segment4' then score5=segment4;
if combo5='Segment5' then score5=segment5;
if combo5='Segment6' then score5=segment6;
if combo5='Segment7' then score5=segment7;
if combo5='Segment8' then score5=segment8;
if combo5='Segment9' then score5=segment9;
if combo5='Segment10' then score5=segment10;
if combo5='Segment11' then score5=segment11;
if combo5='Segment12' then score5=segment12;
run;

data complete;
set complete;
avg_score5=mean(score1,score2,score3,score4,score5);
run;
proc means data=complete noprint;
```

```
var avg_score5;
class id;
output out=out1 mean=mean_5;
run;
data out1;
set out1;
if _n_=1 then delete;
run;
proc means data=complete noprint;
var total_all_12;
class id;
…."
```

*This is an example of the code developed for all combinations of choosing 5 street segments out of the 12, or $_{12}C_{5}$, street segments per address.
